# Irregularities of the Heart

**Published:** 1906-07-21

**Authors:** James Mackenzie

**Affiliations:** Burnley.


### Hospital Clinics.

### IRREGULARITIES OF THE HEART.

By James Mackenzie, M.D., Burnley.

### The Need for Systematic Heart Study.

It is a curious trait of the human mind to attribute the causation of events whose nature is not understood to malignant agencies. From this characteristic has arisen the conception of the demonology of many religions, the dire forebodings with which mysterious but natural events, as comets, are viewed, and the vague speculations which considered certain diseases as the work of evil spirits. With the advance of science it might have been supposed that this tendency to attribute the unknown to malignant agencies would have been avoided, but even in this twentieth century, among the highly cultivated, who have deserved and obtained that hall-mark of true science, the F.R.S., this bent of the human mind still persists. Nowhere is the tendency of this archaic characteristic better seen than in the present-day attitude towards the subject of heart irregularities. Practically little or no close or systematic study has been devoted to this subject, so that writers, when they have to deal with it, treat it in a vague and indefinite manner, and in their conclusions give rein to their imagination. To the human mind the object of dread is always more terrible when it is only partially revealed, so in regard to heart irregularities imperfect knowledge has surrounded it with unnecessary horrors. Many writers hint at grave and hidden dangers, but never specify wherein these dangers lie. This result is reflected upon the practice of the profession in general to such an extent that some simple irregularities are viewed as manifestations of some grave disorder. An individual conscious of his heart's irregularity may thereby be rendered extremely apprehensive. His apprehension is, however, often developed into a morbid dread by the vague and indefinite forebodings of his medical adviser, and the patient's life may be rendered a burden by attempting to carry out the instructions which are supposed to avert the hidden danger of which the heart's irregularity is a forewarning.

### Irregularity not always a Danger.

So far indeed from the irregular action of the heart being an event of some danger, a large proportion of such cases showing irregular action of the heart are in perfect health and are in no danger. The notion that the heart to be normal must be regular is firmly implanted in the medical mind. To remove this false conception it is only necessary carefully to observe the heart of the young at rest, or after a febrile attack. In such circumstances the irregularity of the heart's action is so common that I am persuaded that it is a perfectly natural phenomenon. Again, the action of the heart is so frequently irregular at the other extreme of life that, if it may not be considered normal, it is at all events but one of the many changes that affect the body with advancing years.

Another view of heart irregularity seems to have entirely escaped recognition—namely, where it acts beneficially and saves the organism from some impending calamity. That this last view is a correct one I have now by me many distinct proofs in its favour. On the other hand, that irregularity may be the first sign of an impending failure of the heart is unquestionable. These different views of the meaning of the heart irregularities urgently require consideration in order that one can tell when the danger impends and when there is no cause for uneasiness.

### Necessity for Graphic Records.

In order the more fully to understand the nature of heart irregularities, it is absolutely necessary to take graphic record of the movements of the heart, or other parts of the circulatory system. I hasten to add that in ordinary routine practice this is by no means necessary, provided that the knowledge of graphic records of heart irregularities has been previously acquired. Already in a few schools graphic methods are being employed; and I have no doubt that in a course of 15 or 20 years such methods will be found to be absolutely necessary in all teaching hospitals. Hitherto analyses of the varied forms of irregularity have been left to the imagination of writers with the result that the subject has been overcrowded and confused with a great number of useless and misleading appellations and divisions.

### The Functions of Cardiac Fibres.

To appreciate fully the real nature of heart irregularities the method by which the movements of the heart are carried out should be fully comprehended. Gaskell has demonstrated that the heart muscle fibres possess functions peculiar to themselves. They have the power of creating material capable of producing a stimulus (rhythmicity) the power of becoming susceptible to any stimulus (excitability) the power of conveying the stimulus from fibre to fibre (conductivity), the power of responding to the stimulus by contraction (contractility), and the power of maintaining that slight contraction called tone (tonicity). These five functions are all inherent in the muscle fibres, and can be exercised independently of any nervous stimulation, although nerve action has undoubtedly a modifying effect on them.

In the process of a contraction of the heart all these functions are exercised. The stimulus material excites the fibres which then convey the stimulus to all parts of the heart, and this stimulus at once produces a contraction. Exhaustion immediately follows this exercise of the fibres and all the functions are for the time being abolished, the restoration taking place gradually. This transient abolition of these functions constitutes what is called the refractory period of the heart. Thus immediately after contraction the heart is incapable of being stimulated to further contraction until a certain time has elapsed. When it begins to become susceptible to stimulation, it requires a much stronger stimulus to provoke a contraction than is necessary at a later period—that is to say, that after the refractory period the susceptibility of the heart to stimulation gradually increases. This susceptibility to stimulation under normal circumstances affects all the functions alike. Wenckebach conceived the idea that if one or more functions should not have their susceptibility increased at the normal rate it was probable that certain differences might take place in the execution of the heart's movements, with the result that irregularities might arise. Numerous observations have fully confirmed this prediction and this variation in the rate of the restoration of the functions of the heart muscle fibres is really the key to the interpretation of many forms of heart irregularity, and also supplies the suggestion of rational treatment in many cases of heart failure. When the various agencies, which in the course of a lifetime wear out the heart, such as valvular disease, increased arterial pressure, over exertion, the various infectious and inflammatory affections of the heart muscle begin to interfere with the normal action of the heart it will be found that different parts of the heart and different functions of the heart muscle are unequally affected. One function may be exposed to a greater strain than the other and as a consequence exhaustion of this function will interfere with the rhythmical revolution of all the portions of the heart. Or one portion of the heart muscle may be more or less susceptible to stimulation than another, and this again may seriously modify the heart's rhythm.

### The Various Forms of Irregularity.

The suggestion conveyed in this reference to the heart's functions is fully borne out by the careful study of the various forms of heart irregularity. Thus if the power of conducting the stimulus be impaired in the fibres joining the auricle and ventricle there may result various irregularities due to the stimulus not reaching the ventricle. Other forms of irregularity may arise from varying or undue excitability of certain parts of the heart muscle. It is well known that the contraction of the heart first starts in the muscle fibres at the mouths of the great veins and coronary sinus. The susceptibility of these fibres may vary so that the rhythm of the heart is continually changing in obedience to some varying excitation. Thus in many people the rhythm of the heart varies continually with the respiratory movements. The power of starting the contraction being inherent in all muscle fibres, though in the normal more developed in those at the mouth of the great veins, certain circumstances may excite other fibres to contract before these fibres at the mouths of the great veins. Under such circumstances irregularities due to the ventricle starting the contraction before the auricle can often be recognised. This form of irregularity is extremely common in advanced years and is comparatively rare in the young. It is perhaps the cause of nine-tenths of the irregularities which we meet in elderly people. In the vast majority of cases this form of irregularity leads to no serious result, but may be looked upon as one of the many symptoms associated with advancing years. Its immediate cause, as I have said, is due to the contraction of the ventricle prematurely. While it is difficult to explain the exact method by which this is brought about, one fact stands clearly forth in connection with this irregularity—namely, that it is frequently associated with a temporary increase of the arterial pressure. In many elderly people the arterial pressure has a tendency to rise and to fall. During such a rise the rhythm of the heart may become irregular and this irregularity will be found to be due to the more or less frequent occurrence of these premature ventricular contractions. Under rare conditions these ventricular contractions may be so frequent as to alternate with the normal beat, giving rise to the form of arrhythmia called the *pulsus bigeminus*, or a few of these premature contractions may follow one another, or, again, the rhythm of the heart may start entirely at the ventricle. These observations corroborate in a peculiar manner a series of experiments by H. E. Hering. This experimenter has demonstrated that by raising the intraventricular pressure the ventricle can be made to contract before the auricle, and that occasionally the rhythm of the heart may start from the ventricle.

### How to Value the Irregularities.

It is impossible within the limits of this paper to enter fully into the discussion, either of the physiological production of the various irregularities, or of the clinical significance of the different forms, but as arrhythmia is so often improperly interpreted, I will make a few remarks upon the methods of ascertaining the value of some of the more common irregularities. A very safe, and on the whole satisfactory standard by which one can estimate the significance of any cardiac abnormality, is to estimate the extent of the reserve power latent in the heart. The heart of any individual at rest may be capable of carrying on its work with apparent ease, but on moderate exertion symptoms indicative of heart failure may be provoked, that is to say, there is a great limitation of the field to which the heart can respond. It is really this reserve force which endows the heart with its ability to respond to a varying amount of exertion, and it is this limitation of the field of cardiac response that I employ as the best means of testing the heart's condition, and, consequently, as a means of finding out the real significance of any abnormality. There is no standard for defining the field of cardiac response beyond the individual's own sensations. When we, therefore, find the individual complaining of symptoms which we can attribute to cardiac insufficiency on undertaking such exertion as experience would lead us to expect that he should be able to undertake with comfort, then we recognise that some defect exists, which may have some relationship to any abnormality present. If such abnormality be of the nature of an irregular action of the heart, then we may assume that the cause of the irregularity itself is related to the cardiac weakness. This method of judging the value of the irregularity of the heart's action is applicable to all other symptoms of cardiac abnormalities. Thus, the presence of a murmur at any orifice should not be the sole evidence upon which our diagnosis, prognosis, and treatment should rest. The medical man who for a long period of years intelligently watches the progress of his patient will often be surprised at the manner in which patients with valves of the heart damaged can pursue for years, with perfect freedom, most laborious occupations, free from any heart trouble. Even patients with long-standing mitral stenosis, who may at times have suffered from the most serious attacks of heart-failure, and whose hearts may have become permanently irregular in their action (the so-called mitral pulse) may recover to such an extent as to work as labourers and mechanical engineers with little or no distress. From such observations I feel confident in asserting that we should base our opinion of the heart not upon its abnormality, but upon its capacity for responding to effort, so that when the patient presents himself for examination and exhibits any form of cardiac abnormality, if the patient's field of cardiac response is what one would expect for his years, then we may safely assert that there is very little the matter, and that for the time being, for all practical purposes, he may be considered a healthy man. That the cause of the abnormality may in the long run induce a failure of the heart is a matter which requires careful consideration, but, generally speaking, irregularities with a good field of cardiac response are not of very serious import.

